# Minithoracotomy as a primary alternative for LV lead implantation during coronary resynchronization therapy

**DOI:** 10.1186/1749-8090-8-S1-P104

**Published:** 2013-09-11

**Authors:** S Putnik, N Aleksic, M Matkovic, A Mikic, M Velinovic, VZ Jovicic, I Bilbija, F Vucicevic, D Ivanisevic, M Ristic

**Affiliations:** 1 Cardiac Surgery, Clinical Center of Serbia, Belgrade, Serbia

## Background

Numerous anomalies of cardiac venous system prevent optimal endovascular implantation of LV lead in more than 15% of patients with heart failure and indications for Coronary Resynchronization Therapy (CRT). Insisting on endovenous approach in these patients can be one of the potentional reasons for large number of nonresponders reported in the literature.

Purpose of this study was to analyze the results of an alternative mioepicardial approach to the stimulation of the left ventricle in CRT.

## Results

Between June 2011 to December 2012 in the Department of Cardiac surgery Clinical Centre of Serbia 8 myoepicardial LV leads for CRT were implanted. Coronary sinus venography revealed thrombosis of the coronary sinus in 3 and unfavorable anatomy of the coronary venous system in five patients. In all patients limited left thoracotomy was used as an approach to the lateral wall of the heart. Six patients were in NYHA III and two in NYHA functional class IV preoperative, the average width of QRS complex was 169.7 ± 21 ms, six minute walk test was 284 ± 23.3 m, LV EF was 24.2 ± 4.8%, and MR was 2.16 ± 1.2. There were no major surgical complications and no hospital lethal outcomes. One patient developed hematoma at the site of the surgical incision, which did not require new surgical intervention. In six month follow up period we registered a significant increase in the value of six minute walk test (for an average of 57.9 m), the reduction of QRS complex width (to 26.25 ms), the increase of LVEF (12.2%), and the reduction of MR for 1 +. Based on all the parameters it was concluded that all patients responded favorably to the applied CRT.

## Conclusion

Closer cooperation between cardiologists and cardiac surgeons in identifying a group of patients which would benefit the most from mioepicardial approach for LV stimulation is necessary in order to attempt the reduction of "nonresponder" rate.

